# Supplementary material for: The interactome of intact mitochondria by cross-linking mass spectrometry provides evidence for coexisting respiratory supercomplexes
Source: Mol Cell Proteomics. 2017 Dec 8;17(2):216–32. doi: 10.1074/mcp.RA117.000470 (PMC5795388; doi:10.1074/mcp.RA117.000470)
Supplement: Supplemental Data [file supp_RA117.000470_133922_0_supp_23978_fzffwf.zip › spectra_annotation/mito_DR_spectra_annotation/194-1-18-1-20-1.pdf]

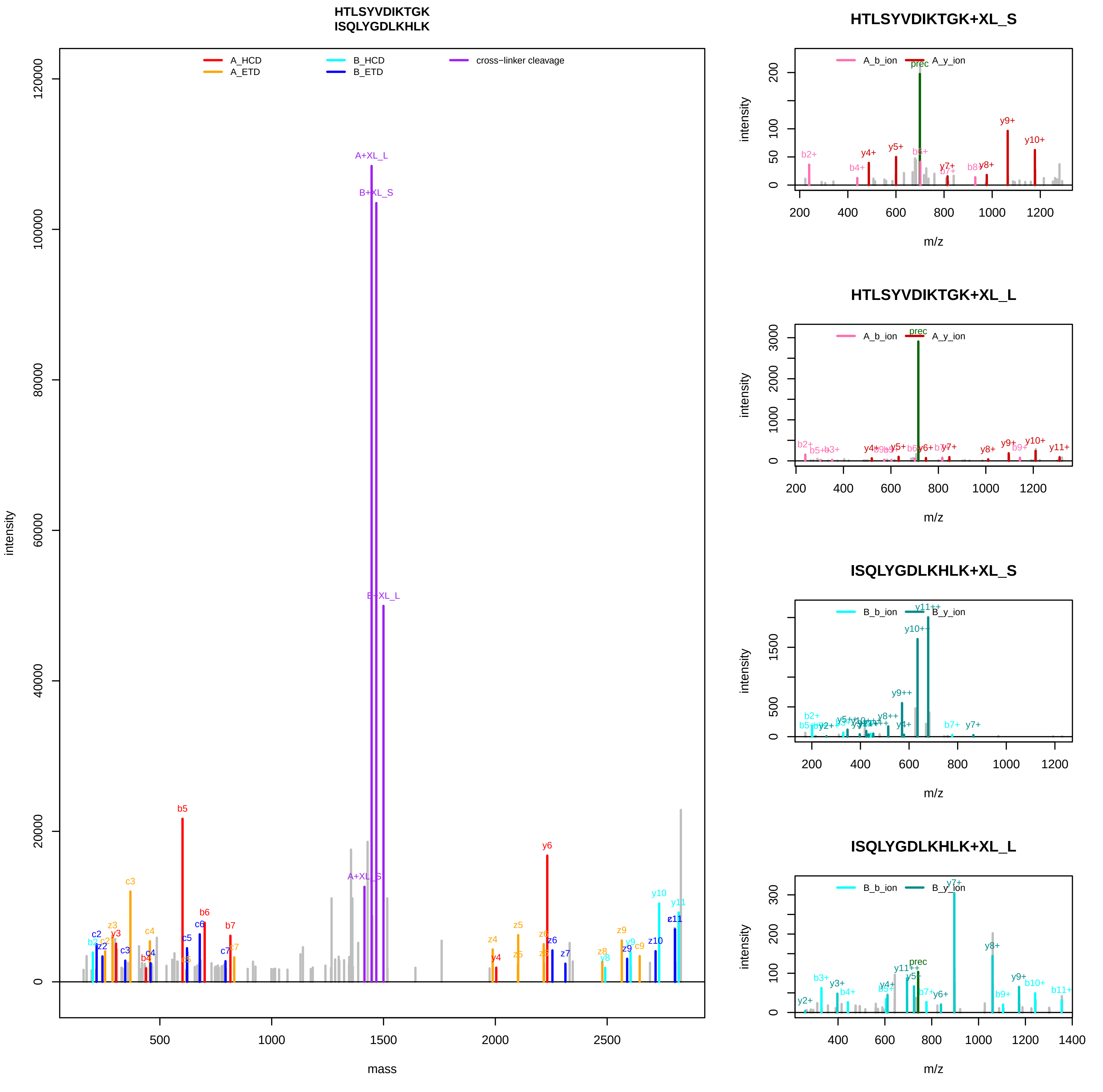
HTLSYVDIKTGK
ISQLYGDLKHLK
A_HCD
A_ETD
B_HCD
B_ETD
cross−linker cleavage
intensity
mass
A+XL_L
B+XL_S
B+XL_L
A+XL_S
b5
y6
b6
b7
c3
c4
c5
c6
c7
z3
y3
c2
z2
b2
b4
y4
z4
z5
z6
z7
z8
y8
z9
y9
c9
z10
y10
y11
z11
HTLSYVDIKTGK+XL_S
HTLSYVDIKTGK+XL_L
ISQLYGDLKHLK+XL_S
ISQLYGDLKHLK+XL_L
A_b_ion
A_y_ion
B_b_ion
B_y_ion
prec
m/z
y9+
y10+
y11+
y11++
y10++
y9++
y8++
y8+
y7+
b7+
b10+
b11+
